# Psychotherapy training of early career psychiatrists: global challenges, opportunities, and future directions

**DOI:** 10.1192/j.eurpsy.2026.10309

**Published:** 2026-07-31

**Authors:** M. Di̇nçer

**Affiliations:** Child and Adolescent Psychiatry, Adnan Menderes University, Aydin, Türkiye

## Abstract

**Abstract:**

Psychotherapy is a core competence in psychiatry, but training experiences among Early Career Psychiatrists (ECPs) remain highly variable across countries, institutions, and supervisors. Some trainees have structured curricula and regular supervision, while others rely on informal mentorship, external courses, or personal effort and finances. This variability is noteworthy where expectations are high and global but pathways are unclear and local. Across settings, recurrent barriers emerge, such as limited protected time, heavy clinical workloads, scarce or inconsistent supervision, unequal access to courses and resources, and financial constraints. Another layer is cultural and institutional context—what is feasible, acceptable, and sustainable in one system may not translate directly to another. At the same time, there are important opportunities. Scalable, low-cost models, Balint groups, structured workshops, and online/hybrid models can broaden access to learning. Competency-based approaches and logbooks can support transparency and standardization.Also, new tools, such as artificial intelligence, may offer additional educational support if used carefully and ethically. This workshop brings together international perspectives to reflect on current practice, and discuss what a feasible, high-quality psychotherapy training pathway for ECPs could look like.

**Disclosure of Interest:**

None Declared

